# *QuickStats:* Percentage* of Adult Day Services Centers^†^ That Use Any Telehealth,^§^ by U.S. Census Bureau Region — United States, 2022^¶^

**DOI:** 10.15585/mmwr.mm7330a4

**Published:** 2024-08-01

**Figure:**
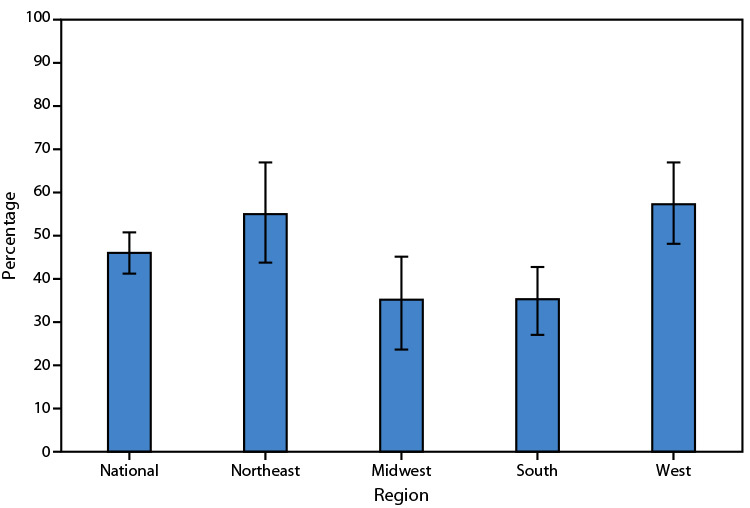
In 2022, 46% of U.S. adult day services centers used any telehealth tools. Approximately one half of centers in the Northeast and West used any telehealth, compared with approximately one third of centers in the Midwest and South.

**Supplementary Table:** https://stacks.cdc.gov/view/cdc/157543

**Source:** National Center for Health Statistics, National Post-Acute and Long-Term Care Study, 2022. https://www.cdc.gov/nchs/npals

